# Abortion and Lamb Mortality between Pregnancy Scanning and Lamb Marking for Maiden Ewes in Southern Australia

**DOI:** 10.3390/ani12010010

**Published:** 2021-12-21

**Authors:** Thomas Clune, Amy Lockwood, Serina Hancock, Andrew N. Thompson, Sue Beetson, Angus J. D. Campbell, Elsa Glanville, Daniel Brookes, Colin Trengove, Ryan O’Handley, Gavin Kearney, Caroline Jacobson

**Affiliations:** 1 Centre for Animal Production and Health, Food Futures Institute, Murdoch University, South Street, Murdoch, WA 6150, Australia; t.clune@murdoch.edu.au (T.C.); a.lockwood@murdoch.edu.au (A.L.); s.hancock@murdoch.edu.au (S.H.); Andrew.Thompson@murdoch.edu.au (A.N.T.); susanbeetson@icloud.com (S.B.); 2 Nossal Institute for Global Health, Melbourne School of Population and Global Health, University of Melbourne, Melbourne, VIC 3010, Australia; a.campbell@unimelb.edu.au; 3 Mackinnon Project, Faculty of Veterinary & Agricultural Sciences, University of Melbourne, Werribee, VIC 3030, Australia; elsa.glanville@unimelb.edu.au (E.G.); dbrookes@unimelb.edu.au (D.B.); 4 School of Animal and Veterinary Science, University of Adelaide, Roseworthy, SA 5371, Australia; colin.trengove@adelaide.edu.au (C.T.); ryan.ohandley@adelaide.edu.au (R.O.); 5 36 Payne Road, Hamilton, VIC 3300, Australia; gke29755@bigpond.net.au

**Keywords:** ewe lamb, hogget, *in utero* loss, pregnancy loss, primiparous, reproduction, sheep, yearling

## Abstract

**Simple Summary:**

The reproductive efficiency of ewes in their first breeding season (maiden ewes) can be inconsistent and disappointing. The frequency of abortion and its relative contribution to lamb losses in maiden ewe flocks in Australia has not been well studied. This study measured abortion and lamb mortality occurring between pregnancy diagnosis (scanning) and lamb marking in 30 flocks of maiden ewes on Australian sheep farms. The study included flocks of ewe lambs that had lambed for the first time at approximately one-year-old (*n* = 19) and two-tooth ewes that had lambed for the first time at two-years-old (*n* = 11). Abortion was detected in 14/19 flocks of ewe lambs and 6/11 flocks of two-tooth ewes using repeated scans. On average 5.7% of ewe lambs and 0.9% of two-tooth ewes aborted; however, abortion rates between flocks ranged from 0–50% for ewe lambs and 0–4.4% for two-tooth ewes. Lamb mortality from birth to marking represented the greatest contributor to overall lamb mortality occurring after pregnancy scanning but abortions were an important contributor to the overall losses in some ewe lamb flocks. This study highlights the variability in reproductive performance for maiden ewes and indicates that addressing losses due to abortion may improve reproductive performance in some ewe lamb flocks.

**Abstract:**

The contribution of abortions to the overall mortality of lambs born to maiden (primiparous) ewes in Australia remains unclear. This cohort study aimed to quantify abortion and lamb mortality for ewe lambs and maiden Merino two-tooth ewes. Lamb mortality from pregnancy scanning to marking were determined for 19 ewe lamb and 11 Merino two-tooth ewe flocks across southern Australia. Average lamb mortality from scanning to marking was 35.8% (range 14.3–71.1%) for the ewe lambs and 29.4% (range 19.7–52.7%) for the two-tooth ewes. Mid-pregnancy abortion was detected in 5.7% of ewes (range 0–50%) in the ewe lamb flocks and 0.9% of ewes (range 0–4.4%) in the two-tooth ewe flocks. Mid-pregnancy abortion affecting ≥2% of ewes was observed in 6/19 ewe lamb flocks and 2/11 two-tooth ewe flocks. Lamb mortality from birth to marking represented the greatest contributor to foetal and lamb mortality after scanning, but mid-pregnancy abortion was an important contributor to lamb mortality in some ewe lamb flocks. Variability between the flocks indicates scope to improve the overall reproductive performance for maiden ewes by reducing foetal and lamb losses. Addressing mid-pregnancy abortion may improve the reproductive performance in some flocks.

## 1. Introduction

The reproductive performance of maiden (primiparous) ewes is an important component of overall flock performance in Australia given maidens represent 20–30% of breeding ewes [1]. Maiden ewes are joined either as ewe lambs at 7 to 10 months of age, or two-tooth ewes, at 18 to 20 months of age. Economic modelling has shown that improving the reproductive performance of ewe lambs and maiden two-tooth ewes are key priorities for improving the reproductive performance of the Australian sheep flock [2]. Lamb mortalities in the perinatal period are a major source of reproductive inefficiency for mature ewe flocks [3]; however, the nature, timing, and magnitude of foetal and lamb mortality between pregnancy scanning and marking is not well studied for maiden ewes.

Some overseas studies have shown that *in utero* losses, including abortions, during mid- to late-pregnancy may be an important contributor to the reproductive inefficiencies of maiden ewes [4,5,6,7,8]. Variable levels of pregnancy loss and abortion are reported for maiden ewes. Some studies in New Zealand have reported abortion in less than 5% of maiden ewes [9,10], whilst others have reported abortions ranging from 5–59% for maiden ewes [4,7,8,11,12,13]. In Australia, abortion events are generally sporadic and considered an insignificant contributor to overall lamb mortality, based on studies conducted using mature or mixed-age ewes [14,15,16,17,18]. A frequency of abortion of 2% is considered ‘normal’, but detection of abortion in sheep managed in extensive production systems is challenging. Furthermore, most Australian studies investigating lamb mortalities between scanning and lamb marking do not distinguish between the losses occurring *in utero* and those in the perinatal period. Anecdotal reports from Australian sheep producers suggest that abortions post-scanning may be contributing to poor reproductive outcomes for maiden ewes, but the relative contribution of abortion to overall lamb mortality for maiden ewes in Australia is unclear.

There are several possible causes of pregnancy loss and abortion between scanning and birth in maiden ewes. Younger ewes appear to be more susceptible to infectious causes of abortion such as campylobacteriosis and toxoplasmosis [19], as they have had less time to develop immunity prior to pregnancy [20]. Lower ewe liveweight at joining and during early pregnancy has also been associated with increased rates of abortion during mid- to late-pregnancy in ewe lamb flocks in New Zealand [11]. Although, this association was not observed in other studies [13]. Multiple factors may be contributing to the *in utero* losses and lamb mortality concurrently in a single flock, making it challenging to identify specific risk factors.

The aims of this study were to (i) quantify abortion and lamb mortality from maiden ewes joined either as ewe lambs or two-tooth ewes, (ii) investigate the timing of abortion and lamb mortalities between pregnancy scanning in mid-pregnancy and lamb marking, and (iii) determine whether the frequency of abortion was associated with ewe liveweight or body condition during pregnancy. We hypothesised that abortion will be a significant contributor to overall mortality between scanning and lamb marking for maiden ewes on commercial farms in Australia.

## 2. Materials and Methods

### 2.1. Research Sites, Animals, and Experimental Design

**Figure 1:**
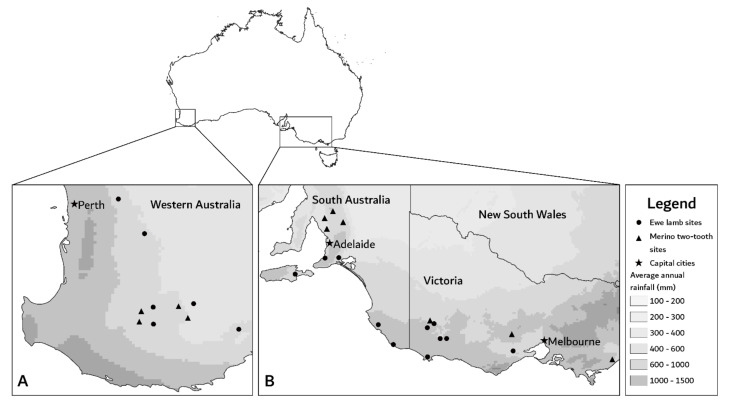
Approximate location of each farm where maiden ewes were monitored in (**A**) Western Australia and (**B**) South Australia and Victoria. Data for average annual rainfall was sourced from Australian Government Bureau of Meteorology [21].

This observational cohort study was conducted using 30 maiden ewe flocks on 28 farms across Western Australia (*n* = 11 flocks), South Australia (*n* = 9 flocks) and Victoria (*n* = 10 flocks) between 2018 and 2020. Farms were located in medium (400–600 mm rainfall annually) to high (600–1000 mm rainfall annually) rainfall zones (Figure 1). The study was performed in consecutive years using different study flocks on two farms; one from Western Australia (2018 and 2019; Flocks 3 and 14) and one from Victoria (2019 and 2020; Flocks 19 and 27). All other farms were sampled in a single year.

Farms were selected on convenience sampling with criteria for inclusion based on: having at least 200 maiden ewes available for the study, a capacity to monitor ewes and their progeny over the study period, and with a sheep genotype and management system that were generally representative of commercial sheep farms in the region. Some farms included in the study managed flocks of stud sheep, but management and stocking density were comparable to commercial sheep flocks in these regions.

Flocks of approximately 200 ewe lambs (*n* = 19 flocks; 7–10 months of age at joining) or Merino two-tooth ewes (*n* = 11 flocks; 18–20 months of age at joining) were monitored from joining to lamb marking on each farm. For farms with more than 200 maiden ewes, a subset of approximately 200 ewes were randomly selected from the larger cohort for inclusion in the study. Ewe lambs were of non-Merino breeds except for one research site (Flock 8) in Western Australia which joined Merino ewe lambs to Merino rams. Most sires were the same breed as the ewes in the study flock; however, White Suffolk rams were used in two flocks joining maiden Merino two-tooth ewes in South Australia (Flock 9 and 12). All rams were confirmed to be negative for *Brucella ovis* via serology prior to joining. Most ewes were naturally joined with a ratio of 50 ewes per ram and joined for an average of 38 days. Some flocks were separated into smaller mobs at joining to facilitate single-sire mating. All or some of the ewes in five flocks were artificially inseminated followed by a period of natural joining.

Each farm ran self-replacing flocks and sheep were managed as per standard farm practice including the monitoring of condition score to guide nutrition and grazing management. Seven of the thirty flocks had been vaccinated against *Campylobacter* spp. (Coopers Ovilis Campyvax^®^, MSD Animal Health, Bendigo, VIC, Australia) according to the manufacturer’s instructions (Table S1). On most farms, the study flock was kept separate from other maiden and mature sheep. Some farms managed their single- and multiple-bearing maiden ewes together in a single mob whilst others separated their single- and multiple-bearing ewes prior to the start of lambing.

### 2.2. Animal Measurements and Sample Collection

The study involved observations and measurements at five timepoints between joining and lamb marking, plus lambing rounds performed by farm staff. Lamb marking was performed approximately 6 weeks from the start of lambing (Table 1).

### 2.3. Liveweight and Condition Score

Liveweight and condition score were recorded for ewes at the five timepoints as outlined in Table 1. Condition score was determined by palpation on a scale of 1 (very thin) to 5 (very fat) as described by Jefferies [22] and van Burgel et al. [23]. All investigators had received training in body condition score assessment and where possible, condition score was assessed by the same person across all time points for each flock.

### 2.4. Assessment of Pregnancy Status and Abortion

Ewes were pregnancy scanned using transabdominal ultrasonography twice during pregnancy (Table 1). Pregnancy scanning for foetal number and viability were performed by experienced researchers, veterinarians or private contractors. On average, the first pregnancy scan (Scan 1) was performed at 85 days from the introduction of rams (range 62–101). The second pregnancy scan (Scan 2) was performed at, on average, 118 days from the introduction of rams (range 107–136) and on average, 33 days (range 21–44) after the first scan. Where possible, scanning was performed by the same operator at Scan 1 and Scan 2 at a single site.

Ewes that were not pregnant, that is, no visible foetus or placentomes detected, were removed from the study after sampling at Scan 1. Pregnancy viability at Scan 2 was confirmed by detection of foetal heartbeats and/or vigorous foetal movements. Loss of pregnancy (i.e., no foetus/es detected) or foetal mortality (i.e., no evidence of foetal viability) between Scan 1 and Scan 2 was validated using lambing records and udder inspection at marking and will be defined as ‘mid-pregnancy abortion’ hereafter. Partial loss of foetuses (e.g., detection of twin pregnancy at Scan 1 and single pregnancy at Scan 2) was not detected, as foetal number was not reliably determined at Scan 2. Therefore, any ewes that may have had partial loss of foetuses were not categorised as mid-pregnancy abortion.

Ewes were checked by farm staff at least twice weekly during pregnancy by observing the ewes in their paddocks. For flocks where mid-pregnancy abortion was detected at Scan 2, the ewes were subsequently checked at least every second day and farm staff were alerted to the possibility of detecting aborted foetuses.

### 2.5. Measurements during the Lambing Period

Farm staff checked the lambing ewes once or twice daily throughout the lambing period. For flocks that tagged lambs at birth (*n* = 19; Table 2), pregnant ewes were assigned a unique paint brand on their sides or fitted with a unique neck tag before lambing to enable the lamb birth type (single, twin or triplet), birth status (dead or alive) and identification to be recorded against the dam identification within 24 h of birth.

Maternal parentage was determined via DNA testing alone in one flock (Flock 10) and in combination with tagging at birth for two flocks (Flock 19 and 27). A combination of tagging at birth and proximity sensors at lamb marking were used to determine the maternal parentage in one flock (Flock 1) [24,25].

Maternal parentage of lambs was not determined for eight flocks (Table 2). In seven of these flocks, pregnant ewes were assigned a unique paint brand on their sides or fitted with a unique neck tag before lambing. Lamb birth type and birth status were recorded against the dam by observation and ewe lactation status was assessed at marking to determine if the ewe was rearing a lamb. For these flocks, the total number of lambs born per flock was estimated based on the number of lambs present at marking plus the number of dead lambs collected or observed during the lambing period. The number of lambs born may have been underestimated because it is unlikely that all lambs that died were observed (e.g., lost to predation). For Flock 24, lambing rounds were not performed, but a third pregnancy scan was performed at the pre-lambing visit (134 days from the start of joining) to determine if pregnancy losses had occurred since Scan 2 and these results were used as a proxy for the number of lambs born.

Lambs that were dead at the lambing rounds (i.e., died between birth and tagging) were categorised as ‘born’ and therefore were included in lamb mortalities between birth and marking. For flocks where lambs were tagged at birth, identification of live lambs was recorded at lamb marking to determine the lamb survival to marking for individual ewes. Some dead lambs could not be allocated to a ewe but were included in the total count of the number of lambs born at the flock-level. It is possible that some lambs that died were not recovered during the lambing rounds and therefore the number of lambs born may be underestimated for some flocks.

Ewe udders were assessed at lamb marking to determine their lactation status using the categories lactating (wet) or non-lactating (dry). Ewes that failed to lamb were identified using lambing records (no lamb/s allocated to ewe) and ewe lactation status at marking (dry). Rear type, or number of lambs reared to marking per ewe, was determined by recording the ear tag identification of lambs present at marking and validated using a ewe udder assessment. Ewe mortalities during the lambing period were recorded by the farm staff during daily inspections.

### 2.6. Lamb Cause of Death and Detection of Infectious Disease

Lambs that died in the first three days following birth were retained for necropsy to determine the cause of death as described in detail and reported elsewhere by Clune et al. [26]. Tissue samples from aborted (*n* = 2) or stillborn (*n* = 33) lambs recovered from a subset of eight flocks (Flocks 1, 2, 3, 7, 11, 14, 16 and 19) were tested for evidence of infectious disease using microbial culture and/or molecular diagnostics as reported by Clune et al. [26]. Ewe serology were performed for *Toxoplasma gondii* [27], *Campylobacter* spp. [28], *Neospora caninum* [29] and *Coxiella burnetii* [30] and reported elsewhere.

### 2.7. Quantitative Variables

Conception rate (%) was calculated for each flock as the number of ewes pregnant at Scan 1 divided by the number of ewes present at joining. The scanning rate (%) was calculated for each flock as the number of foetuses identified at Scan 1 divided by the number of ewes present at joining.

Mid-pregnancy abortion frequency (%) was calculated using either the number of foetuses that were lost or not viable at Scan 2 expressed as a proportion of foetuses detected at Scan 1, or the number of ewes with evidence of abortion at Scan 2 expressed as a proportion of ewes determined to be pregnant at Scan 1.

Foetal and lamb mortality between Scan 1 and marking were calculated using the number of foetuses identified at Scan 1, the viability of pregnancy at Scan 2, the number of lambs born and marked per ewe, and lactation status.

### 2.8. Statistical Analyses

Data from Flock 6 and 9 (maiden Merino two-tooth ewe flocks) and Flock 25 (ewe lamb flock) were incomplete (Tables S1 and S2). Data from these flocks were included in descriptive statistics, where appropriate, but excluded from analyses using generalised linear mixed models (GLMM) where data for the outcome variable (mid-pregnancy abortion or overall lamb mortality) were incomplete.

Data were analysed by the following methods using GENSTAT (VSN International 2017). Estimates of mid-pregnancy abortion between Scans 1 and 2 were assessed by fitting GLMM. The approach used a logit transformation and binomial distribution. Using additive models, logits were predicted as a function of birth type (scanned single or multiple), liveweight and condition score at joining and interactions thereof as fixed effects, while flock was fitted as a random effect. Estimates of overall mortality from Scan 1 to marking were assessed by fitting GLMM. The approach used a logit transformation and binomial distribution. Using additive models, logits were predicted as a function of birth type, liveweight and condition score at joining, liveweight and condition score change from joining to Scan 1 and interactions thereof as fixed effects, while flock was fitted as a random effect.

Lamb mortality (%) between Scan 1 and Scan 2, Scan 2 and birth and birth and marking within flocks were compared using a 2-tailed Chi-square test. Statistical significance was accepted where *p* ≤ 0.05.

## 3. Results

### 3.1. Mid-Pregnancy Abortion—Ewe Lambs

A total of 2968 pregnant maiden ewe lambs from 19 flocks were included in this study. Mid-pregnancy abortion was observed in 14/19 (73.7%) flocks (Table S1). The frequency of mid-pregnancy abortion was ≥2% of ewes in 6/19 (31.6%) flocks (Table S1). The frequency of mid-pregnancy abortion ranged from 2.1–50% for ewes in these flocks, representing 3.4–48.4% of foetuses identified at Scan 1 (Table 3).

Mid-pregnancy abortion accounted for 12.4% of overall lamb mortality between Scan 1 and marking; however, there was considerable between-flock variation (Table S1). The majority (63.6%) of mid-pregnancy abortions were observed on four farms (Flock 3, 14, 19 and 24) in which mid-pregnancy abortion accounted for 16.4–68.1% of the total foetal and lamb mortalities observed between Scan 1 and marking (Table S1).

Visual evidence of abortion such as aborted foetuses, retained foetal membranes, vaginal discharge or staining of the perineal region or hindlegs before the expected lambing date were noted for five flocks. Aborted foetuses were recovered from only three flocks, all of which were between Scan 2 and the start of lambing (Table S1).

Mid-pregnancy abortion was observed in both Flock 3 (6.2% foetuses; 2018) and Flock 14 (21.7% foetuses; 2019) which were located on the same farm (Table S1). For the other farm that was sampled over consecutive years, mid-pregnancy abortion was observed in Flock 19 (8.6% foetuses, 2019), but not Flock 27 (2020). Campylobacteriosis was diagnosed in Flock 19 by microbial culture, and vaccination for *Campylobacter* spp. was implemented by the farmer for Flock 27 in 2020.

### 3.2. Timing of Foetal and Lamb Mortality—Ewe Lambs

Timing for abortion and lamb mortality in the ewe lamb flocks are shown in Table 3. On average, 35.8% of foetuses identified at Scan 1 failed to survive to lamb marking (Table 3). The relative contribution of foetal or lamb mortality within each time period varied between flocks (Table S1). Lamb mortality between birth and marking was the largest contributor to lamb mortality for most farms (Table S1). Ewe mortalities (*n* = 28) during the lambing period resulted in mortality for 0.83% of foetuses (*n* = 38) identified at Scan 1.

### 3.3. Factors Associated with Foetal and Lamb Mortality—Ewe Lambs

Liveweight and condition score for single- and multiple-bearing ewe lambs are shown in Table S3. Average liveweight and body condition score at joining for single-bearing ewes were 48.0 kg and 3.3, respectively, and for multiple-bearing ewes were 49.8 kg and 3.4, respectively (Table S3). Liveweight and condition score at joining, and liveweight change from joining to Scan 1, had no effect on mid-pregnancy abortion (*p* > 0.05), nor on overall lamb mortality between scanning and marking (*p* > 0.05).

Lamb birth type (litter size) had no effect on mid-pregnancy abortion (Table 4). The overall lamb mortality between scanning and marking was lower for lambs scanned as singles (24.5%) compared to lambs scanned as multiples (twins or triplets; 31.2%) (*p* < 0.001).

### 3.4. Mid-Pregnancy Abortion—Merino Two-Tooth Ewes

A total of 1886 pregnant maiden Merino ewe two-tooth ewes from 11 flocks were included in this study. Mid-pregnancy abortions were detected in 6/11 (54.5%) flocks (Table S2) and accounted for the mortality of 0.8% foetuses (Table 3). Mid-pregnancy abortion contributed 2.8% of the overall lamb mortality between Scan 1 and marking.

The frequency of mid-pregnancy abortion was ≥2% of ewes in 2/11 (18.2%) flocks where abortion frequency ranged from 2.2–4.4% of pregnant ewes and 1.7–3.7% of foetuses identified at Scan 1 (Table S2). No aborted foetuses were recovered from the two-tooth ewe flocks. Blood staining on the hindlegs of two ewes was observed in Flock 13 where 0.9% of ewes had mid-pregnancy abortion, but no visual evidence of abortion was reported in other flocks.

### 3.5. Timing of Foetal and Lamb Mortality—Merino Two-Tooth Ewes

On average, 29.4% of foetuses identified at Scan 1 failed to survive to lamb marking for the Merino two-tooth ewes (Table 3). As with the ewe lambs, the relative contribution of foetal or lamb mortality within each time period varied between flocks (Supplementary Table S2). Lamb mortality between birth and marking was the greatest contributor to foetal and lamb mortality (Table S2). Ewe mortalities (*n* = 7) during the lambing period resulted in the mortality of 0.35% foetuses (*n* = 8) identified at Scan 1.

### 3.6. Factors Associated with Foetal and Lamb Mortality—Merino Two-Tooth Ewes

Liveweight and condition score for single- and multiple-bearing maiden Merino two-tooth ewes are shown in Table S3. The average liveweight and body condition score at joining for single-bearing ewes were 48.6 kg and 2.9, respectively, and for multiple-bearing ewes were 49.2 kg and 3.0, respectively (Table S3). Liveweight and condition score at joining, and liveweight change from joining to Scan 1 had no effect on mid-pregnancy abortion (*p* > 0.3), nor on overall foetal and lamb mortality between scanning and marking (*p* > 0.05).

Mid-pregnancy abortion was 1.63% greater for single foetuses compared to multiple foetuses (*p* = 0.022; Table 4); however, the overall mortality between Scan 1 and marking for lambs scanned as multiples was greater than lambs scanned as singles (47.4% vs. 30.3%; *p* < 0.001).

## 4. Discussion

Pregnancy scanning rates for maiden ewes and mortality of their lambs between pregnancy scanning and lamb marking varied widely between flocks. Lamb mortality was greatest between birth and marking for the maiden Merino two-tooth ewe flocks and most flocks of ewe lambs; however, abortion during mid- and late-pregnancy were significant contributors to overall lamb mortality for some ewe lamb flocks. Therefore, our hypothesis that abortion will be a significant contributor to lamb mortality between scanning and lamb marking for maiden ewes is partially accepted. Over 50% of foetuses identified at pregnancy scanning subsequently aborted or died in some flocks, representing substantial production and economic losses for these enterprises; however, evidence from other flocks in our study shows that mortality below 20% is achievable for lambs born to ewe lambs and maiden Merino two-tooth ewes in southern Australia. Our observations indicate that strategies which reduce perinatal lamb mortality should be prioritised for improving lamb survival for maiden ewes; however, identifying and addressing mid-pregnancy abortions may improve reproductive performance in some ewe lamb flocks. The causes of mid-pregnancy abortion are poorly understood and further investigation of the pathophysiology and economic losses associated with mid- and late-pregnancy abortion are warranted. This will inform cost-benefit analyses for interventions to address these losses.

The frequency of mid-pregnancy abortion was greater than the widely accepted ‘normal’ level of abortion of 2% for one third of ewe lamb flocks. The mean frequency of mid-pregnancy abortion of 5.7% across ewe lamb flocks was consistent with the apparent 9% foetal mortality between scanning and birth for non-Merino ewes up to 18 months of age at lambing recorded on the Maternal Sheep Genetics Australia database (Daniel Brown, pers. comms). It is also consistent with studies from New Zealand which reported that the frequency of abortion ranged from 3.7–8.1% in commercially managed ewe lamb flocks based on sequential scanning performed at similar timepoints to our study [11,13]. In contrast to ewe lambs, the mean frequency of mid-pregnancy abortion was less than 1% for Merino two-tooth ewe flocks, making mid-pregnancy abortion a minor contributor to the overall lamb mortality in these flocks. This was consistent with an Australian study reporting an apparent 3% foetal mortality between scanning and birth in mixed age ewes, although this may have included lambs that died soon after birth but were not identified at lambing rounds [15].

Mid- to late-pregnancy abortion was largely inconspicuous, even in flocks with a high mid-pregnancy abortion frequency detected by scanning. Evidence of abortion such as an aborted foetus, retained foetal membranes or bloodstaining of the hind legs was only observed in five ewe lamb flocks. In most cases this was detected by research staff because of additional monitoring of sheep occurring as part of the study. It is likely that some foetal mortality during mid-pregnancy resulted in resorption of the foetus. Otherwise, paddock topography, predation, and the length of pasture in good seasons make it challenging for farmers to detect aborted foetuses or foetal membranes during the inspection of flocks, particularly given the small size of the foetus with a mid-pregnancy abortion. Disease investigation of abortion cases is more likely to result in aetiological diagnosis where placental tissue is available [19]. For flocks where mid-pregnancy abortion is suspected, farmers should be advised to be extra vigilant to identify aborting ewes, and where possible, collect material to rule out infectious causes.

Errors in assessing the foetal number at scanning may contribute to errors in the estimation of abortion and lamb mortality between scanning and marking. In this study, the use of sequential pregnancy scans was an effective strategy for determining mid-pregnancy abortion given that complete pregnancy loss between Scan 1 and Scan 2 was the principle measure used to identify ewes with mid-pregnancy abortion. However, the frequency of abortion, including late-pregnancy abortion, may be underestimated using only two scans at the timepoints used in this study. Further, distinguishing late-pregnancy abortion from perinatal lamb deaths is challenging on extensively managed sheep farms due to issues recovering dead lambs related to predation and paddock characteristics. A third pregnancy scan (after day 130 of pregnancy) identified late-pregnancy abortion (pregnancy loss between Scan 2 and pre-lambing) in some ewes that had viable pregnancies at Scan 2 for Flock 24. Therefore, a later second scan or third scan may help to quantify *in utero* losses and distinguish these from perinatal losses in flocks with evidence of mid-pregnancy abortion. The decision to use additional scans to identify flocks with mid-pregnancy abortions should consider the costs associated with an additional scan and the risk of handling multiple-bearing ewes in late gestation.

Mid-pregnancy abortion was not associated with either liveweight or condition score at joining or liveweight change in early pregnancy. This is in agreeance with studies in ewe lambs in New Zealand under pastoral conditions [31,32,33]. By contrast, Mulvaney et al. [34] and Ridler et al. [11] reported that a higher frequency of abortion was associated with lower liveweight at joining and lower liveweight gain in early pregnancy. High liveweight gains during early pregnancy were also associated with foetal mortality in the study by Mulvaney et al. [34]. However, the overall average pre-joining liveweight in this study (48 kg for single-bearing ewes and 49.8 kg for multiple-bearing ewes) was higher than the pre-joining liveweight of ewe lambs in the study by Mulvaney et al. [34] (36 kg) which may explain why we observed no association between liveweight at joining and mid-pregnancy abortion. Whilst standardised methods were used for the measurement of body condition score, assessment is subject to operator bias [23]. Calibration of body condition score measurement between assessors could have reduced between-operator variability in body condition score assessment. The general linear mixed model included farm as a random effect and therefore adjusted for the condition score assessor because condition score assessor was confounded with farm. Other studies have shown experienced assessors are able to achieve a high degree of consistency even when using 0.25 score units [35].

The overall lamb mortality in ewe lamb flocks (36%) was within the range of 19–43% observed for the progeny of ewe lambs on commercial farms in New Zealand [8,36]. There was marked variation between farms and even between years on the same farms joining ewe lambs as per Thompson et al. [36], however, this variation was not explained by liveweight at joining, liveweight profile during early pregnancy or lamb birth type. Inconsistent reproductive performance in ewe lambs has been identified as a barrier to the adoption of joining ewe lambs [37]. Whilst ewe lamb reproductive performance can be improved by optimising the ovulation and reproductive rate by liveweight at joining [38,39,40], observations from this study indicate that efforts to reduce foetal and lamb mortality between scanning and marking will also be valuable in improving the reproductive output of ewe lamb flocks.

The average overall lamb mortality between scanning and marking in maiden two-tooth ewe flocks in this study (29%) was comparable to other studies reporting a 33% lamb mortality in Merino two-tooth ewe flocks [41], and within the lamb mortality range of 25–30% reported for mixed age Merino ewes [16]. As with ewe lambs, the reproductive performance of maiden Merino two-tooth ewes can be improved with interventions to optimise fertility and fecundity [17]; however, findings from our study suggest that there is scope to improve maiden two-tooth ewe reproductive performance with interventions that improve lamb survival, and especially in the period between birth and marking.

Most lamb deaths occurred at or after birth. Lamb necropsies were conducted on a subset of farms and are reported elsewhere [26]. Dystocia, stillbirths, and starvation-mismothering accounted for most mortalities which is consistent with other Australian studies [3,42]. Higher rates of mortality were observed for multiple-born lambs which is consistent with previous observations for predominantly mature ewe flocks [17,43,44,45,46]; however, there was considerable variation in lamb mortality between birth and marking between flocks in this study. This may be explained by differences in genetics, environmental factors and other management factors (e.g., mob size and supplementary feeding) on lamb survival [3,43,47,48]. The use of lamb necropsies to identify cause of death can be used to inform targeted strategies for improving perinatal lamb survival. Conducting necropsies also provides opportunity for the detection of infectious diseases that may be associated with abortion or poorer lamb viability. Identification of factors impacting lamb survival in the perinatal period informs targeted strategies that can improve the reproductive performance of maiden flocks.

We did not identify any relationship between the ewe condition score or liveweight at joining or the liveweight profile and overall foetal and lamb mortality in ewe lamb flocks. This was consistent with other recent work reporting that liveweight at joining and liveweight profile may have relatively little impact on the mortality of lambs born to ewe lambs [36]. A range of other factors could have contributed to abortion and perinatal mortality. Stillbirths, dystocia and starvation-mismothering accounted for the majority of perinatal mortalities identified by gross pathology for the subset of flocks for which lamb necropsies were performed [26]. These causes of perinatal mortality are often multifactorial and related to genetic, environmental and management factors [3,48]. Sporadic impacts of endemic diseases could also explain the between-flock variation in lamb mortality and absence of relationship with ewe liveweight or liveweight profile. There was evidence that endemic diseases were contributing to abortions and stillbirths in some flocks in this study. For example, abortions, stillbirths and polyarthritis associated with *Chlamydia pecorum* were identified in a subset of flocks in this study [26,49], and campylobacteriosis (*Campylobacter fetus* fetus) was identified in one flock [28]. There was no evidence that infection with *Toxoplasma gondii* [27], *Neospora caninum* [29] or *Coxiella burnetii* [30] were important contributors to foetal and lamb mortalities in these flocks. Younger ewes are more susceptible to some endemic diseases because they are less likely to have developed immunity through previous infection. Disease investigations are warranted for maiden ewe lamb flocks with disappointing or inconsistent lamb survival to inform targeted strategies addressing lamb survival. Further studies to improve the understanding of the causes of mid- and late-pregnancy abortion in Australian ewes will inform targeted strategies addressing abortion and lamb survival.

## 5. Conclusions

Lamb mortality between scanning and lamb marking is highly variable for maiden ewe flocks. Perinatal lamb deaths represent the most important source of reproductive loss between scanning and marking in most maiden ewe flocks; however, significant post-scanning *in utero* losses occur due to mid-pregnancy abortion in some flocks. Liveweight or condition score at joining or liveweight change in early pregnancy had no effect on mid-pregnancy abortion or overall lamb mortality in these flocks. Sequential pregnancy scanning can be used to identify mid-pregnancy abortion and differentiate *in utero* losses from perinatal mortality. Disease investigations are warranted for maiden ewe flocks with evidence of abortion or disappointing lamb survival. Differentiating the losses associated with mid- and late-pregnancy abortion from perinatal losses, and determination of aetiological diagnosis where infectious disease is implicated will inform strategies to improve the reproductive performance and lamb survival in maiden ewe flocks.

## Figures and Tables

**Table 1:** Summary of timepoints, corresponding stage of the reproductive cycle and measurements performed throughout the study period for each flock.

Timepoint	Stage of Reproductive Cycle	Measurements
1. Joining	Approx. 0–14 days prior to joining	Ewes: CS and LW
2. First pregnancy scan (Scan 1)	Average of 85 days from the start of joining (range 62–101 days)	Ewes: CS, LW and pregnancy ultrasound for foetal number
3. Second pregnancy scan (Scan 2)	Average of 118 days from the start of joining (range 107–136 days)	Ewes: CS, LW and pregnancy ultrasound for foetal viability
4. Pre-lambing	Average of 138 days from the start of joining	Ewes: CS and LW
5. Lambing	Lambing period	Lambs: number of lambs born to each ewe, birth status of lambs (dead/alive) and total number of lambs born ^
6. Lamb marking	Approx. 6 weeks from the start of lambing	Ewes: CS and LW Lambs: number of lambs marked to each ewe and total number of lambs marked ^

CS: condition score, LW: liveweight. ^ Lambs were not tagged at birth for some flocks. In these flocks, number of lambs born per ewe was determined by distance observation and ewe lactation status was used to determine if the ewe reared a lamb.

**Table 2:** The number of flocks which determined birth type and rear type of lambs born to ewe lambs and maiden two-tooth ewes by tagging at birth, DNA testing at marking, tagging at birth and using sensors for maternal pedigree at marking, and those using other methods to assess lamb birth type, lamb birth status and whether ewes were rearing a lamb.

Method of Determining Birth Type, Rear Type, and Maternal Parentage of Individual Lambs	Ewe Lambs (*n*)	Two-Tooth Ewes (*n*)
Tagging at birth	13	5
DNA testing at marking	2	1
Tagging at birth and sensors for maternal pedigree at marking	0	1
Other ^	4	4

^ Individual lamb birth type, rear type and maternal parentage not determined. The total number of lambs born was estimated using a count of live lambs at marking plus the number of dead lambs recovered, or via a pregnancy scan at the pre-lambing timepoint. The number of lambs born per ewe was determined by distance observation. Ewe lactation status was used to determine whether ewes were rearing a lamb.

**Table 3:** Overall reproductive performance and timing of foetal and lamb mortalities for 19 flocks of ewe lambs and 11 flocks of maiden Merino two-tooth ewes across southern Australia between 2018 and 2020.

	Ewe Lambs		Two-Tooth Ewes	
	Mean	Range	Mean	Range
Conception rate (%) ^1^	73.4	45.4–92.4	87.1	58.5–97.1
Scanning rate (%) ^2^	112.7	57.7–155.6	104.7	59.6–135.2
**Mid-pregnancy abortion**				
% foetuses ^3^	5.5	0–48.4	0.8	0–3.7
% ewes ^4^	5.7	0–50.0	0.9	0–4.4
**Foetal loss between scan 2 and birth** (% foetuses) ^5^	10.5	0–27.5	10.3	0–40.2
**Lamb mortality between birth and marking** (% lambs) ^6^	18.0	8.7–28.1	19.0	10.6–26.0
**Overall foetal/lamb mortality between Scan 1 and marking** (% foetuses)	35.8	14.3–71.1	29.4	19.7–52.7

^1^ Number of ewes pregnant at Scan 1/number of ewes joined (%), ^2^ Number of foetuses identified at Scan 1/number of ewes joined (%), ^3^ Number of foetuses lost between Scan 1 and Scan 2/number of foetuses identified at Scan 1 (%), ^4^ Number of ewes with foetal loss between Scan 1 and Scan 2/number of ewes joined (%), ^5^ Number of foetuses present at Scan 2 but not accounted for at lambing/number of foetuses identified at Scan 1 (%). Includes lambs that were born and not recovered at lambing rounds (i.e., lost to predation). ^6^ Number of lambs that died between birth and marking/number of foetuses identified at Scan 1 (%). Includes lambs dead at birth (full-term).

**Table 4:** Transformed and back-transformed means for frequency of mid-pregnancy abortion in maiden ewe lambs and Merino two-tooth ewes determined using a general linear mixed model.

	Mid-Pregnancy Abortion Frequency Transformed Data (Back Transformed Mean) ^1^	
Litter Size	Ewe Lambs	Two-Tooth Ewes
Single	−4.21 (1.46%)	−3.91 (1.98%)
Multiples	−4.37 (1.25%)	−5.67 (0.34%)
Standard error of the difference	0.256	0.771

^1^ Number of foetuses aborted between Scan 1 and Scan 2/number of foetuses detected at Scan 1 (%).

## Data Availability

The datasets generated and/or analysed during the current study are not publicly available but are available from the corresponding author on reasonable request pending permission from the funding body (Meat and Livestock Australia).

## Supplementary Materials

The following are available online at https://www.mdpi.com/article/10.3390/ani12010010/s1, Table S1: Reproductive performance and timing of abortion and lamb mortalities for 19 flocks of ewe lambs across southern Australia between 2018 and 2020; Table S2: Reproductive performance and timing of abortion and lamb mortalities for 11 flocks of maiden Merino two-tooth ewes across southern Australia between 2018 and 2020; Table S3: Mean of flock means for liveweight and body condition score for single- and multiple-bearing ewes from ewe lamb and maiden Merino two-tooth ewe flocks in southern Australia between 2018 and 2020.
