# Supplementary material for: Abortion and Lamb Mortality between Pregnancy Scanning and Lamb Marking for Maiden Ewes in Southern Australia
Source: Animals (Basel). 2021 Dec 21;12(1):10. doi: 10.3390/ani12010010 (PMC8749747; doi:10.3390/ani12010010)
Supplement: Supplementary file 1 [file animals-12-00010-s001.zip › animals-1471938-supplementary.pdf]

animals

MDPI

Supplementary Materials

Abortion and Lamb Mortality between Pregnancy Scanning and Lamb Marking for Maiden Ewes in Southern Australia

Thomas Clune [1], Amy Lockwood [1], Serina Hancock [1], Andrew N. Thompson [1], Sue Beetson [1], Angus J.D. Campbell [2], Elsa Glanville [3], Daniel Brookes [3], Colin Trengove [4], Ryan O'Handley [4], Gavin Kearney [5] and Caroline Jacobson [1,*]

[1] Centre for Animal Production and Health, Food Futures Institute, Murdoch University, South Street, Murdoch, WA 6150, Australia; t.clune@murdoch.edu.au (T.C.); a.lockwood@murdoch.edu.au (A.L.); s.hancock@murdoch.edu.au (S.H.); Andrew.Thompson@murdoch.edu.au (A.T.); susanbeetson@icloud.com (S.B.)

[2] Nossal Institute for Global Health, Melbourne School of Population and Global Health, University of Melbourne, VIC 3010, Australia; a.campbell@unimelb.edu.au

[3] Mackinnon Project, Faculty of Veterinary & Agricultural Sciences, University of Melbourne, Werribee, VIC 3030, Australia; elsa.glanville@unimelb.edu.au (E.G.); dbrookes@unimelb.edu.au (D.B.)

[4] School of Animal and Veterinary Science, University of Adelaide, Roseworthy, SA 5371, Australia; colin.trengove@adelaide.edu.au (C.T.); ryan.ohandley@adelaide.edu.au (R.O.)

[5] Biometrics Consultant, 36 Payne Road, Hamilton, VIC 3300, Australia; gke29755@bigpond.net.au

* Correspondence: C.Jacobson@murdoch.edu.au

Table S1. Reproductive performance and timing of abortion and lamb mortalities for 19 flocks of ewe lambs across southern Australia between 2018 and 2020

**Flock reference no.**	**3**	**4**	**7**	**8**	**11**	**14**	**16**	**17**	**18**	**19**	**20**	**21**	**22**	**23**	**24**	**25**	**27**	**28**	**30**
State	WA	WA	WA	WA	WA	WA	WA	VIC	VIC	VIC	VIC	VIC	SA	SA	SA	SA	VIC	VIC	SA
Breed	NM	NM	NM	Mo	NM	NM	NM	NM	NM	NM	NM	NM	NM	NM	NM	NM	NM	NM	NM
Campylobacter vaccination[1]	No	No	No	No	No	No	No	Yes	Yes	No	No	Yes	Yes	No	Yes	No	Yes	Yes	No
Ewes joined (*n*)	198	200	198	189	213	197	162	196	197	203	204	206	195	201	196	302	200	202	388
Conception rate (%)[2]	74.7	65	76.3	45.5	68.5	85.3	63.6	54.1	85.8	87.2	80.4	76.7	72.3	81.6	62.2	92.4	86.5	78.7	57.7
Scanning rate (%)[3]	97.5	106.5	122.2	57.7	74.6	110.2	91.4	78.6	146.7	143.3	124.0	124.3	123.6	120.9	96.9	155.6	155.5	133.2	79.4
Marking rate (%)[4]	60.6	82.0	88.4	38.6	54.5	45.2	60.5	67.3	117.3	70.9	73.0	75.7	91.8	99.0	28.1	30.1	115.0	94.6	49.5
Aborted foetuses recovered (*n*)	1	0	0	0	0	0	0	0	0	3	0	0	0	0	1	0	0	0	0
**Foetal and lamb mortality (% foetuses)[5]**																			
Overall mortality (scan 1 – marking)	37.8	23.0	27.7	33.0	27.0	59.0	33.8	14.3	20.1	50.5	41.1	39.1	25.7	18.1	71.1	65.6	26.0	29.0	37.7
Mid-pregnancy abortion (scan 1 – scan 2)[5]	6.2	1.4	1.2	1.8	0.0	21.7	3.4	0.0	0.0	8.6	4.3	0.0	0.8	2.1	48.4	1.3	0.0	1.1	1.3
Late-pregnancy loss (scan 2 – birth)[6]	6.2	6.1	17.8	16.5	8.8	9.2	8.8	0.0	3.1	27.5	11.1	25.8	2.1	4.9	7.9	NA	4.8	10.8	16.9
Lamb mortality (birth – marking)[7]	25.4	15.5	8.7	14.7	18.2	28.1	21.6	14.3	17.0	14.4	25.7	13.3	22.8	11.1	14.7	NA	21.2	17.1	19.5
Chi-square *P*-value	<0.001	<0.001	<0.001	<0.01	<0.001	<0.001	<0.001	<0.001	<0.001	<0.001	<0.001	<0.001	<0.001	<0.001	<0.001	NA	<0.001	<0.001	<0.001
**Mid-pregnancy abortion (ewes)**																			
n	11	2	1	1	0	40	3	0	0	15	7	0	1	3	61	4	0	3	3
%	7.4	1.5	0.7	1.2	0.0	23.8	2.9	0.0	0.0	8.5	4.3	0.0	0.7	1.8	50.0	1.4	0.0	1.9	1.3

NA: not available WA: Western Australia SA: South Australia VIC: Victoria NM: non-Merino Mo: Merino

[1] Vaccination with Ovilis Campyvax™ (*Campylobacter fetus* and *Campylobacter jejuni*) [2] Number of ewes pregnant / number of ewes joined [3] Number foetuses scanned / number of ewes joined

[4] Number of lambs marked / number of ewes joined [5] Number of foetuses (or lambs) lost from start to end of respective period indicated in parentheses / number of foetuses at scan 1 (%).

[6] Includes lambs that were born and not recovered at lambing rounds (e.g. lost to predation). [7] Includes lambs dead at birth (full-term)

Table S2. Reproductive performance and timing of abortion and lamb mortalities for 11 flocks of maiden Merino two-tooth ewes across southern Australia between 2018 and 2020.

**Flock reference no.**	**1**	**2**	**5**	**6**	**9**	**10**	**12**	**13**	**15**	**26**	**29**
State	WA	WA	SA	SA	SA	WA	SA	VIC	WA	VIC	VIC
Campylobacter vaccination[1]	No	No	No	No	No	No	No	No	No	No	No
Ewes joined (n)	213	188	194	197	202	200	197	188	210	190	191
Conception rate (%)[2]	87.3	94.7	94.8	83.2	95.5	84.5	92.9	58.5	97.1	72.1	93.1
Scanning rate (%)[3]	116.9	116.5	109.3	90.9	113.9	89.5	113.7	59.6	135.2	85.3	121.5
Marking rate (%)[4]	93.9	81.4	79.4	NA	89.1	66.0	81.2	28.2	101.0	52.1	93.2
**Foetal and lamb mortality (% foetuses)[5]**											
Overall mortality (scan 1 – marking)	19.7	30.1	27.4	NA	21.7	26.3	28.6	52.7	25.4	38.9	23.3
Mid-pregnancy abortion (scan 1 – scan 2)[5]	0.0	0.0	0.9	0.0	0.0	0.0	0.9	0.9	0.7	3.7	1.7
Late-pregnancy loss (scan 2 – birth)[6]	0.0	4.1	3.8	NA	NA	15.6	3.1	40.2	1.8	21.0	3.0
Lamb mortality (birth – marking)[7]	19.7	26.0	22.6	NA	NA	10.6	24.6	11.6	22.9	14.2	18.5
Chi-square *P*-value	<0.001	<0.001	<0.001	NA	NA	<0.001	<0.001	<0.001	<0.001	<0.001	<0.001
**Mid-pregnancy abortion (ewes)**											
n	0	0	2	0	0	0	2	1	1	6	4
%	0.0	0.0	1.1	0.0	0.0	0.0	1.1	0.9	0.5	4.4	2.2

NA: not available WA: Western Australia SA: South Australia VIC: Victoria

[1] Vaccination with Ovilis Campyvax® (*Campylobacter fetus* and *Campylobacter jejuni*)

[2] Number of ewes pregnant / number of ewes joined

[3] Number foetuses scanned / number of ewes joined

[4] Number of lambs marked / number of ewes joined

[5] Number of foetuses (or lambs) lost from start to end of respective period indicated in parentheses / number of foetuses at scan 1 (%).

[6] Includes lambs that were born and not recovered at lambing rounds (e.g. lost to predation).

[7] Includes lambs dead at birth (full-term)

Table S3. Mean of flock means for liveweight and body condition score for single- and multiple-bearing ewes from ewe lamb and maiden Merino two-tooth ewe flocks in southern Australia between 2018 and 2020.

	Mean liveweight and body condition score (range)			
	Ewe lamb		Merino two-tooth ewes	
	Single	Multiple	Single	Multiple
**Liveweight (kg)**				
Joining [1]	48.0 (39.7 - 65.6)	49.8 (40.4 - 67.4)	48.6 (37.5 - 67.5)	49.2 (42.3 - 67.8)
Scan 1 [1]	50.5 (41.0 - 65.6)	53.5 (41.9 - 67.6)	50.8 (37.9 – 73.0)	53.8 (49.3 - 75.1)
Scan 2 [2]	51.0 (34.2 - 68.5)	56.3 (36.3 - 72.6)	51.9 (38.4 - 68.3)	56.1 (48.0 - 69.3)
Pre-lambing [2]	55.4 (39.7 - 77.6)	61.9 (41.9 - 82.9)	55.9 (41.0 - 76.5)	61.9 (52.1 – 78.0)
**Body condition score**				
Joining [1]	3.3 (2.4 – 4.0)	3.4 (2.4 - 4.0)	2.9 (2.6 - 3.6)	3.0 (2.6 - 3.5)
Scan 1 [1]	3.4 (2.8 – 4.0)	3.4 (2.7 – 4.0)	2.9 (2.6 - 3.6)	3.0 (2.6 - 3.4)
Scan 2 [2]	3.3 (2.6 - 3.9)	3.3 (2.7 – 4.0)	2.8 (2.0 - 3.6)	2.9 (2.5 - 3.7)
Pre-lambing [2]	3.3 (2.7 - 4.1)	3.4 (2.6 - 4.1)	2.8 (2.5 - 3.5)	2.8 (2.5 - 3.5)

[1] Flocks 6 and 9 excluded from analyses; [2] Flocks 6, 9 and 25 excluded from analyses.
